# ﻿Descriptions of two new Idiocerini leafhoppers of the genus *Idioscopus* (Hemiptera, Cicadellidae) from China

**DOI:** 10.3897/zookeys.1095.76731

**Published:** 2022-04-13

**Authors:** Xian-Yi Wang, Jia-Jia Wang, Ren-Huai Dai, Mick D. Webb

**Affiliations:** 1 Institute of Entomology, Guizhou University; The Provincial Key Laboratory for Agricultural Pest Management Mountainous Region, Guiyang, Guizhou 550025, China Guizhou University Guiyang China; 2 Department of Life Sciences (Entomology), The Natural History Museum, Cromwell Road, SW7 5BD, London, UK Department of Life Sciences (Entomology), The Natural History Museum London United Kingdom

**Keywords:** Checklist, *Myrica*, taxonomy

## Abstract

Two new species of the leafhopper genus *Idioscopus* Baker are described from China: *Idioscopusbihamulus***sp. nov.** and *I.ventrispinus***sp. nov.**, the latter recorded on a species of *Myrica* L. (Myricaceae) as its host plant. A key and checklist to species of the genus from China are provided and *Idioscopustaiwanus* Huang & Maldonado-Capriles, 1992 is placed as a junior synonym of *Idioscopusclypealis* (Lethierry, 1889), **syn. nov.**

## ﻿Introduction

The leafhopper genus *Idioscopus* was described by Baker, 1915, with *I.clypealis* (Lethierry, 1889) as its type species. Subsequently, many new species of the genus were described by Pruthi (1936), Maldonado-Capriles (1964, 1974), Viraktamath (1976, 1979a, 1979b), Kuoh and Fang (1985), Huang and Maldonado Capriles (1992), and Wang and Dai (2018). Xue et al. (2017) and Wang and Dai (2018) provided keys as well as checklists to Chinese Idiocerinae, including the genus *Idioscopus*. Many species of *Idioscopus* are important agricultural and forest pests; their known hosts are mainly *Mangifera* spp. (Anacardiaceae), *Dimocarpus* sp. (Sapindaceae), *Prunus* sp. (Rosaceae), and *Myrica* sp. (Viraktamath 1989; Khatri and Webb 2014; Wang and Dai 2018). At present, *Idioscopus* comprises more than 30 species of which 10 species are recorded from China (see Checklist). Another species recorded from China by Zhang and Li (2012: 208, pl. 7), i.e., *Idioscopusbimaculatus* (Pruthi, 1936) is misidentified (C. Viraktamath pers. comm.). In this paper, we describe and illustrate two new species of *Idioscopus* from Yunnan Province, China, and provide a revised key and checklist to species from China. In addition, *Idioscopustaiwanus* Huang & Maldonado-Capriles, 1992 is placed as a junior synonym of *Idioscopusclypealis* (Lethierry, 1889), syn. nov., and the identities of two other Idiocerinae from Taiwan, i.e., *Idiocerusapicalis* Matsumura and *I.formosanus* Matsumura are discussed and photographs of the habitus of their types taken by Masami Hayashi in 1995 are provided.

## ﻿Materials and methods

The specimens examined were collected from Yunnan Province, China, using a sweep net. Techniques for the preparation of the genital structures follow Oman (1949) and morphological terminology mainly follows Dietrich (2005). All specimens examined are deposited in the Institute of Entomology, Guizhou University, Guiyang, China (**GUGC**) and The Natural History Museum, Department of Life Sciences (Entomology), Cromwell Road, SW7 5BD, London, UK.

## ﻿Taxonomy

### ﻿Genus Idioscopus

Taxon classificationAnimaliaHemipteraCicadellidae

Baker, 1915

5B441A28-7712-5C06-B90B-D38D77E73772

#### Type species.

*Idiocerusclypealis* Lethierry, 1889 by original designation.

#### Diagnosis.

The genus *Idioscopus* can be distinguished from other genera of Idiocerini by the combination of the following features: style with slender setae on dorsal margin, subapex wide and flat on later on lateral view, aedeagal shaft with one or two pairs of processes.

#### Description.

Body small (♂ 3.10–5.50 mm; ♀ 3.30–5.50 mm). Head wider than pronotum. Head and thorax shagreen or crown and frontoclypeus dorsad of ocelli finely transversely rugose. Face as long as wide to slightly longer than wide; frontoclypeus with lateral margins extending to just above antennae; anteclypeus broad distally, longer than wide, sometimes exceeding apex of gena; rostrum in some species variably expanded apically; ocelli placed closer to midline than to the corresponding eye. Length of visible mesonotum nearly as long or longer than pronotum and crown together. Forewing with four apical and usually two (open or closed) subapical cells. Hind femur with 2 + 1 apical setae.

***Male genitalia*.** Male pygofer in lateral view triangular its height more than its width; dorsoposterior lobe differentiated by dorsoanterior vertical cleft; dorsal anal collar present joined to pygofer; anal tube comprising a single segment, short or long; with or without a basiventral process. Subgenital plate elongate, curved dorsad, with long hair-like marginal setae. Connective Y- or T-shaped, short. Style elongate, apophysis curved dorsally, inner margin crenulate or dentate, outer margin distally with a row of hair-like setae or with a tuft of stout setae. Aedeagus with shaft elongate, either slightly curved dorsally or sinuate, laterally compressed, with one or two pairs of apical processes, gonopore apical on ventral surface; dorsal apodeme well developed, apically expanded laterally.

#### Distribution.

African, Oriental, and Palaearctic regions.

##### ﻿Checklist of *Idioscopus* species from China

(See Xue et al. 2017 for complete synonymy)

*I.bihamulus* sp. nov.

*I.clypealis* (Lethierry, 1889: 252, *Idiocerus*)

*I.taiwanus* Huang & Maldonado-Capriles, 1992: 5–6, syn. nov.

*I.furcaprocessus* Wang, Wang, Zhou & Dai, 2021: 376, fig. 1

*I.longiprocessus* Wang, Wang, Zhou & Dai, 2021: 378, fig. 2

*I.myrica* Wang & Dai, 2018: 12–13, figs 1–10

*I.nitellicus* Kuoh & Fang, 1985: 190, figs 8–16

*I.nitidulus* (Walker, 1869: 322, *Iassus*)

*Idiocerusniveosparsus* Lethierry, 1889: 160; Matsumura, 1912: 322 (Taiwan)

*I.recurvatus* Kuoh & Fang, 1985: 189, figs 1–7

*I.serratastylus* Wang, Wang, Zhou & Dai, 2021: 378, fig. 3

*I.ventrispinus* sp. nov.

### ﻿Key to the species of *Idioscopus* from China

**Table:** 

1	Forewing with yellow patch on clavus (Fig. 2A)	**2**
–	Forewing without yellow patch on clavus	**4**
2	Aedeagus with a pair of subapical processes and a single ventral process (Fig. 2H)	***I.ventrispinus* sp. nov.**
–	Aedeagus with a pair of subapical processes, without a single ventral process	**3**
3	Aedeagus with processes directed laterally	***I.furcaprocessa***
–	Aedeagus with processes directed ventrally	***I.serratastylus***
4	Clypellus dark brown	***I.clypealis***
–	Clypellus not dark brown	**5**
5	Aedeagus with a single pair of apical processes and a single dorsal subapical process	***I.myrica***
–	Aedeagus with one or two pair of subapical processes, without a single dorsal process	**6**
6	Aedeagus with one pair of distal processes	**7**
–	Aedeagus with two pair of distal processes	**8**
7	Aedeagus with processes strongly curved (Fig. 1H, I)	***I.bihamulus* sp. nov.**
–	Aedeagus with processes weakly curved	***I.longiprocessus***
8	Aedeagus with shaft evenly curved in lateral view	***I.nitidulus***
–	Aedeagus with shaft sinuate in lateral view	**9**
9	Aedeagus with one very long and one short subapical processes	***I.nitellicus***
–	Aedeagus with two pair of moderately long subapical processes	***I.recurvatus***

### ﻿ Idioscopus bihamulus sp. nov.

Taxon classificationAnimaliaHemipteraCicadellidae

C3EC927F-8948-57EA-98BA-DD36CB3F7004

http://zoobank.org/8049B242-A15D-408D-8E57-C3F95F0856D8

Figs 1 , 3 A–D

#### Type material.

***Holotype***: ♂, Baoshan City, Mt Gaoligongshan, Yunnan Province, China (98°47'42"E, 25°18'20"N; 1745 m elev.), 4 August 2018, coll. Zhou Yu (GUGC). ***Paratypes***: 1♂, 6♀♀, same data as holotype.

#### Diagnosis.

The new species can be distinguished from other *Idioscopus* species by the combination of the following features: anal tube short; style apophysis with a row of three or four subapical teeth along inner margin; aedeagal shaft with long, recurved, hook-like apical processes.

#### Description.

**Figure 1.**
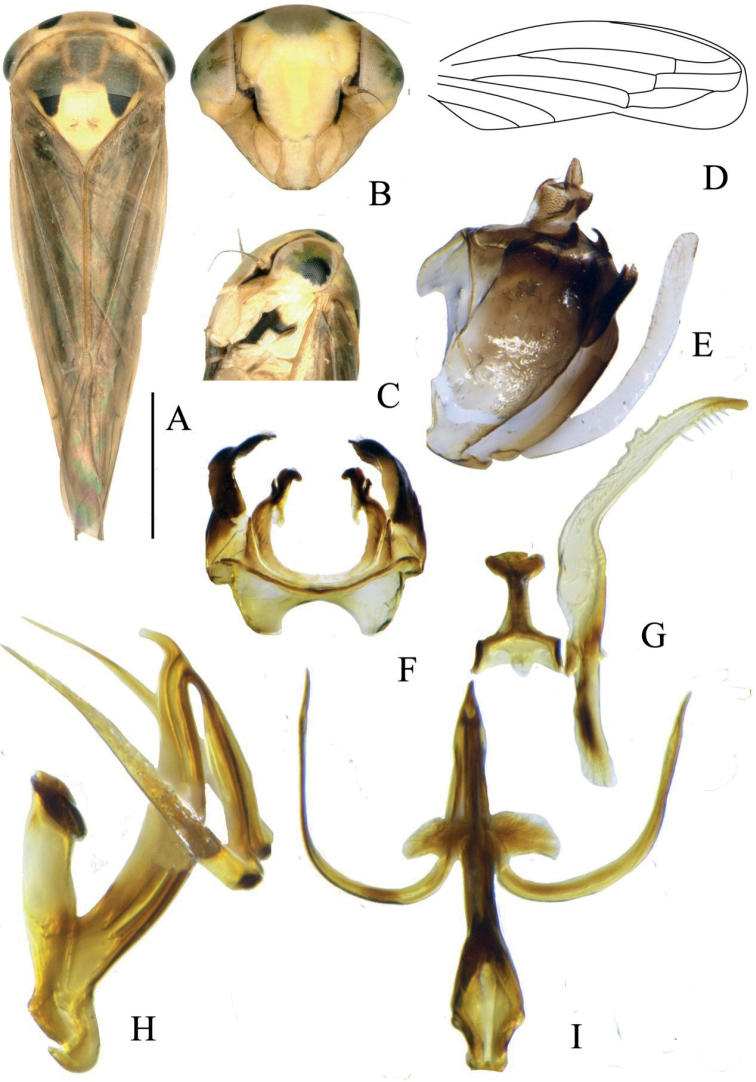
*Idioscopusbihamulus* sp. nov. male **A** habitus, dorsal view **B** face **C** head and thorax, lateral view **D** forewing **E** genital capsule, lateral view **F** pygofer dorsal view **G** connective and style, dorsal view **H** aedeagus, lateral view **I** aedeagus ventral view. Scale bar: 1.0 mm.

***Coloration*.** Ground coloration translucent brown. Vertex pale brown with two round spots close to the adjacent eyes (Fig. 1A). Eyes and ocelli brown (Fig. 1A–C). Face (Fig. 1B, C) yellowish white, narrow area along inner margin of eye, area surrounding bases of antennae, and inner margins of gena along frontal suture black, upper area with dark brown semicircular marking. Pronotum (Fig. 1A) yellowish brown along anterior margin, posterior two-thirds brown, with a brown U-shaped band medially. Mesosternum (Fig. 1A) black, mesepimeron with black spot. Mesonotum (Fig. 1A) yellow with pair of triangular, laterobasal, black maculae. Forewing with veins brown (Fig. 1A).

External features as in generic description with face and pronotum shagreen and crown finely transversely rugose. Male antennae without apical disc. Forewing (Fig. 1D) with two subapical cells; inner subapical open.

**Figure 2.**
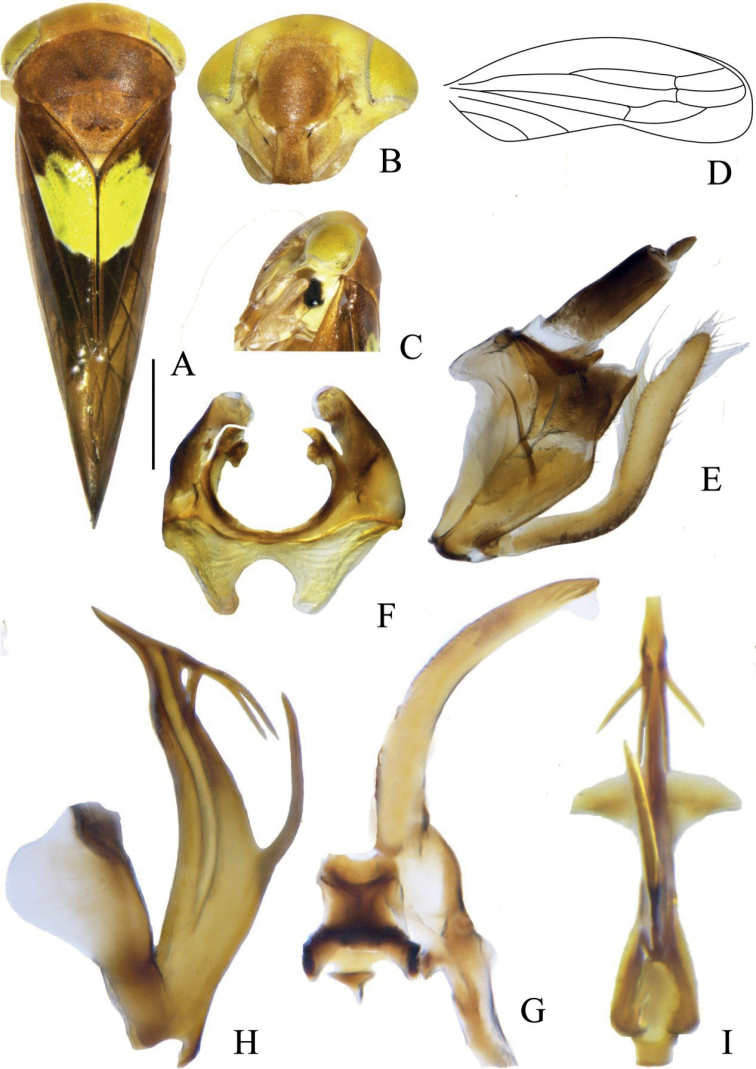
*Idioscopusventrispinus* sp. nov. male **A** habitus, dorsal view **B** face **C** head and thorax, lateral view **D** forewing **E** genital capsule, lateral view **F** pygofer dorsal view **G** connective and style, dorsal view **H** aedeagus, lateral view **I** aedeagus ventral view. Scale bar: 1.0 mm.

***Male genitalia*.** Male pygofer (Fig. 1E, F) without ventral process; anal collar thin with apex upturned; anterodorsal apodemes well developed. Anal tube (Fig. 1E) short. Subgenital plates (Fig. 1E) of uniform width, with reduced hair-like marginal setae. Connective (Fig. 1G) T-shaped, with stem narrow. Style apophysis (Fig. 1G) with a row of three or four tooth-like projections along inner margin subapically, outer margin with macrosetae in distal one-third. Aedeagus (Fig. 1H, I) with shaft laterally compressed, with pair of long, recurved, hook-like processes subapically from ventral margin, acute apically; basal apodeme pillar-like in lateral view, distally and laterally expanded in ventral view.

**Figure 3.**
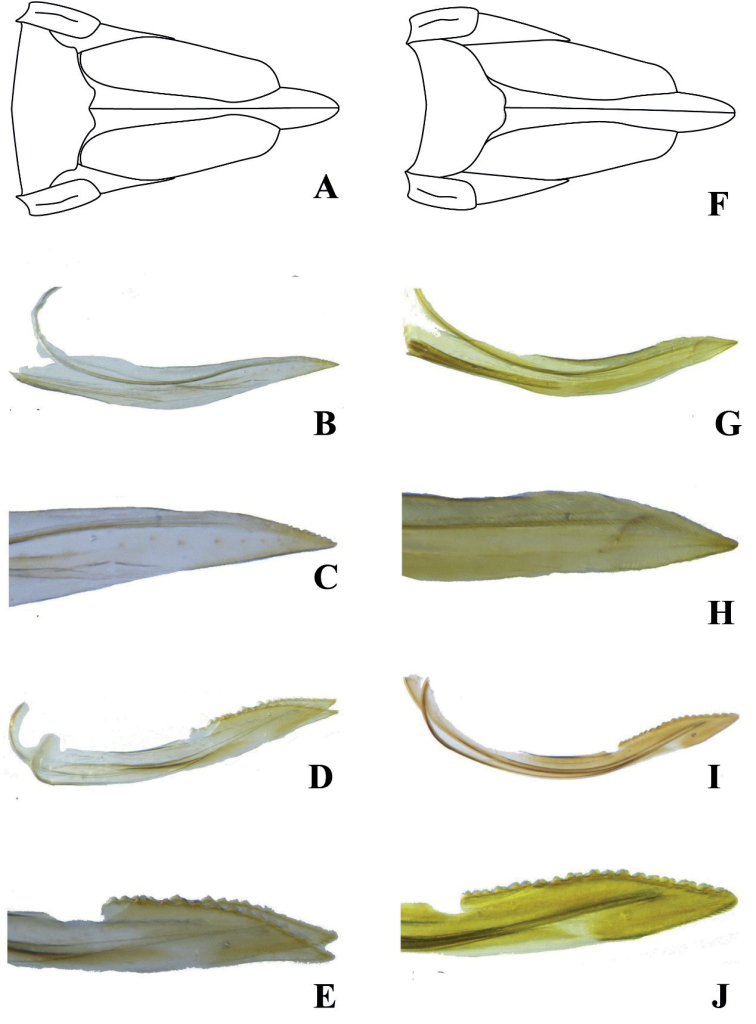
Ovipositor. Female. **A–E***Idioscopusbihamulus* sp. nov. **F–J***Idioscopusventrispinus* sp. nov. **A, F** sternite VII **B, G** first valvula **C, H** apex of first valvula **D, I** second valvula **E, J** apex of second valvula.

***Female genitalia*.** Posterior margin of sternite VII (Fig. 3A) slightly produced medially. Valvulae as in Figure 3B–E.

#### Measurements (mm).

Male: body length including tegmina 3.70–3.80. Medial length of crown 0.13–0.16, width 1.25–1.30. Distance from ocellus to eyes 0.15–0.20. Medial length of pronotum 0.40–0.45, width 1.10–1.20; scutellum length 0.50–0.55, width 0.95–1.05. Female: body length including tegmina 3.70–3.90.

#### Etymology.

The specific epithet refers to the two hamulus-like (hook-like) aedeagal processes.

#### Host plant.

Unknown.

#### Distribution.

China (Yunnan).

### ﻿ Idioscopus ventrispinus sp. nov.

Taxon classificationAnimaliaHemipteraCicadellidae

EFDBCB3E-BA55-52DB-AC49-610149BEA014

http://zoobank.org/F0FFE31A-7C3B-48FD-93F8-F6E3DCB62537

Figs 2 , 3E–H

#### Type material.

***Holotype***: ♂, Xinping County, Mt Ailaoshan, Yunnan Province, China (101°57'57"E, 24°07'39"N; 1956 m elev.), 22 July 2018, coll. Xianyi Wang & Jiajia Wang. ***Paratypes***: 58♂♂ 2♀♀, same as holotype. 4♂♂, Lvchun County, Mt Hoanglianshan, Yunnan Province, China (102°17'27"E, 22°56'03"N; 1815 m elev.), 7 June 2019, coll. Jiajia Wang & Chao Zhang. 1♂, Baoshan City, Mt Gaoligongshan, Yunnan Province, China (98°48'03"E, 25°18'15"N; 1581 m elev.), 22 May 2019, coll. Jiajia Wang & Chao Zhang.

#### Diagnosis.

This new species resembles *Idioscopusfurcaprocessus* in general appearance, including having a yellow patch on the forewing clavus. The sinuate shaft of the aedeagus in lateral view is similar to *I.confuscous* (Pruthi) (see Viraktamath, 1980: fig. 15), *I.bihamulus*, *I.recurvatus*, and *I.nitellicus*, but it differs from these and other species in having an unpaired, ventral, spine-like process proximad of the midlength of the shaft.

#### Description.

***Coloration*.** General color reddish brown. Crown yellowish with darker reddish-brown markings; eyes yellowish (Fig. 2A–C). Pronotum and mesonotum (Fig. 2A) reddish brown, mesonotum paler with yellowish hue. Face (Fig. 2B) greenish yellow with frontoclypeus and anteclypeus pale reddish brown; male antennae with apical disc, black. Mesepimeron (Fig. 2C) with black patch. Forewing clavus (Fig. 2A) with large, lemon-yellow patch bordered with dark reddish brown.

External features as in generic description with head and thorax shagreen; male antennae with apical disc (Fig. 2A–C); forewing (Fig. 2D) with two subapical cells, inner subapical open.

***Male genitalia*.** Male pygofer (Fig. 2E, F) conically rounded apically, without ventral process; anal collar thin, upturned apically; dorsoanterior apodemes well developed. Anal tube (Fig. 2E) long. Subgenital plates (Fig. 2E) broader in distal one-third in lateral view, longer than pygofer, sharply upturned in basal third, with long hair-like marginal setae distally. Connective (Fig. 2G) Y-shaped, broad, with short stem. Style apophysis (Fig. 2G) narrowed to apex with weakly serrated inner margin. Aedeagus (Fig. 2H, I) with shaft laterally compressed, broad in basal half then narrowed and slightly sinuate distally to acute apex in lateral view, with one pair of subapical bifid processes, each fork unequal, an unpaired ventral spine-like process proximad of midlength; dorsal apodeme laterally compressed, distally expanded in lateral view and laterally expanded distally in ventral view.

***Female genitalia*.** Posterior margin of sternite VII (Fig. 3F) slightly produced medially. Valvulae as in Figure 3G–J.

#### Measurements (mm).

Male: body length including tegmina 4.50–4.95. Medial length of crown 0.18–0.22, width 1.55–1.60. Distance from ocellus to eyes 0.15–0.20. Medial length of pronotum 0.45–0.48, width 1.35–1.45; scutellum length 0.50–0.55, width 0.65–0.70. Female: body length 4.60–5.20 including tegmina.

#### Etymology.

The new species name is derived from the words *ventri*- and *spinus*, referring to the ventral spine-like process of the aedeagal shaft.

#### Host plant.

*Myrica* sp.

#### Distribution.

China (Yunnan).

### ﻿ Idioscopus clypealis

Taxon classificationAnimaliaHemipteraCicadellidae

(Lethierry)

772A662D-3CB6-525F-974E-EAAF25A6EA27

Fig. 4

Idiocerus clypealis Lethierry, 1889: 252—Matsumura 1912: 322 (Taiwan).
Idioscopus clypealis —Maldonado Capriles 1964: 92–93, figs 6–9; Khatri and Webb 2014: 282–284, table 1, fig. 5.
Idioscopus taiwanus Huang & Maldonado-Capriles, 1992: 7, fig. 3, syn. nov.

#### Remarks.

**Figure 4.**
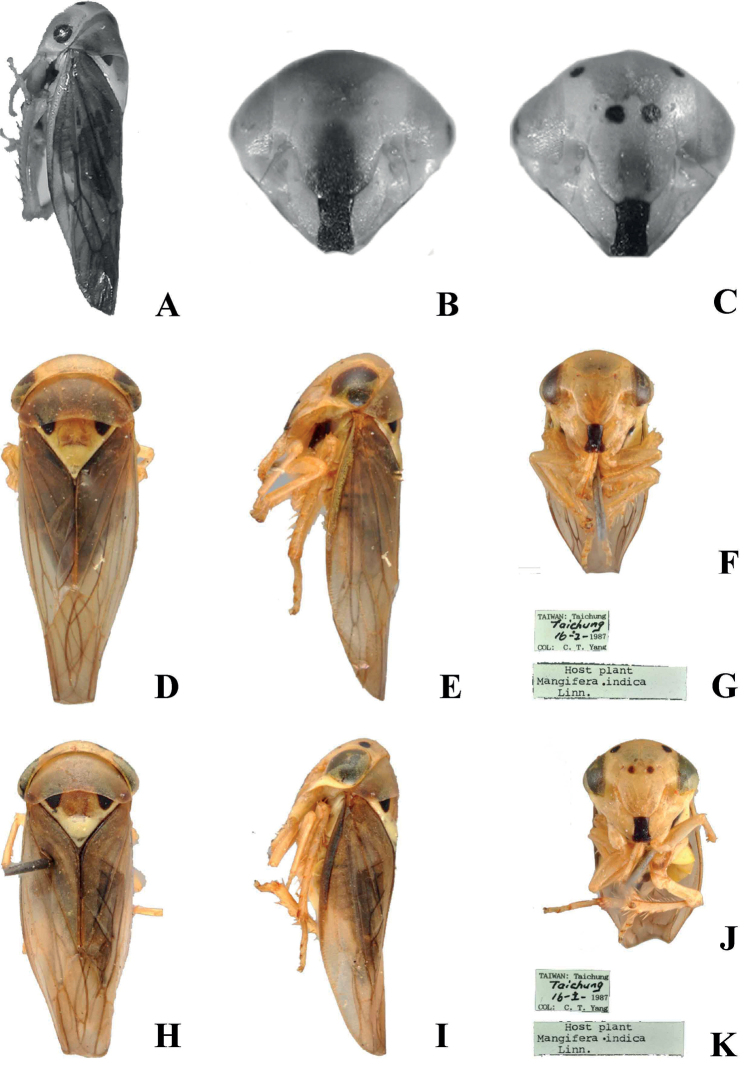
*Idioscopusclypealis***A–C** female (China) **A** lateral habitus female **B, C** face (male and female respectively) showing atypical marking of clypeus; photographs by Xue Qingquan **D–G** paratype male of *I.taiwanus***D** dorsal habitus **E** lateral habitus **F** ventral habitus **G** labels **H–K** paratype female of *I.taiwanus***H** dorsal habitus **I** lateral habitus **J** ventral habitus **K** labels; photographs by David Redei.

The above synonymy of *I.taiwanus* with *I.clypealis* is based on the similarity of the published figures of both species and examination of some paratypes of *I.taiwanus* (see below). It is surprising that *I.taiwanus* was described as a new species and not recognized as the widespread *I.clypealis*, as the latter species was well-known to Maldonado-Capriles and had been earlier figured by him (Maldonado-Capriles 1964). Also, the described colour of *I.taiwanus* more or less matches the “typical” colour form of *I.clypealis* figured by the same author and latter figured by Khatri and Webb (2014) from mainland Asia. A variation of this colour is seen in some specimens collected from mainland China which have more extensive brown marking medially on the face (Fig. 4A–C), matching some Pacific material figured by Khatri and Webb (2014).

*Idioscopustaiwanus* was described from the holotype and 80 paratypes with the following data: “Taiwan, Taichung, 16/1/1987, C. T. Yang collector. Paratypes: 40 males and 40 females, same collection data as holotype. Host plant: *Mangiferaindica*” (Huang & Maldonado-Capriles 1992). In the introduction to their paper, Huang & Maldonado-Capriles stated that the type material was in the “Department of Entomology, National Chung-Hsing University (NCHU), Taichung; Division of Collection and Research, National Museum of Natural Science (NMNS), Taichung; and in the Department of Applied Zoology, Taiwan Agriculture Research Institute (TARI), Wufeng, Taichung.” They added that “Some paratypes deposited in the junior author’s collection”. For other new species described in the same paper the holotype depository is given as NMNS, but no depository is given for *I.taiwanus*; however, the male genitalia dissection of the holotype is present in NMNS (Jing-Fu Tsai pers. comm.) and paratypes are present in both NCHU (see Fig. 4D–K) and NMNS but not in TARI or Maldonado-Capriles’s collection (National Museum of Natural History, Washington, DC, USA).

### ﻿ Idiocerus apicalis

Taxon classificationAnimaliaHemipteraCicadellidae

Matsumura

AA5CC3C7-3300-551F-86F2-FEDAF36298E5

Fig. 5A, B

Idiocerus apicalis Matsumura, 1912: 323—Huang and Maldonado-Capriles 1992: 4 (listed); Xue et al. 2017: 407 (listed).

#### Remarks.

**Figure 5.**
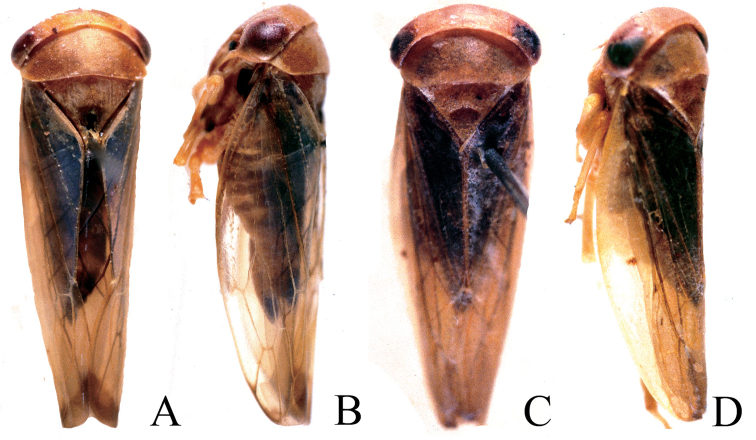
Matsumura Taiwan Idiocerine species **A, B***Idiocerusapicalis*, dorsal and lateral, habitus respectively (holotype) **C, D***I.formosanus*, dorsal and lateral, habitus respectively (syntype). Photographs by Masami Hayashi.

This species was described from a single female specimen (holotype) from Taiwan. Both Andy Hamilton and Masami Hayashi (pers. comm.) examined the type (Entomological Institute, Hokkaido University, Sapporo, Japan) and the latter’s photographs of the type are reproduced here (Fig. 5A, B). The holotype bears a determination label by Andy Hamilton indicating the species belongs to *Balocerus* but this needs to be confirmed, particularly as the specimen is female. The label information on the specimen is as follows (hw = handwritten, pr = printed):

1. *I.apicalis* sp. nov. (hw), det. Matsumura (pr)
2. Formosa, Matsumura (printed), (underside) Toroen, 19/4 ‘07 (hw)
3. Holotype (red label, printed), *Idiocerusapicalis* Matsumura (hw)
4. *Balocerusapicalis* (Matsum.), Det. KGAH ‘76 (hw; label with red frame)

### ﻿ Idiocerus formosanus

Taxon classificationAnimaliaHemipteraCicadellidae

Matsumura

0AC53060-9EB6-5088-9D92-F35DBEAE4160

Fig. 5C, D

Idiocerus taiwanus Matsumura—Huang and Maldonado-Capriles 1992: 4 (listed); Xue et al. 2017: 407 (listed).

#### Remarks.

This species was described from an unknown number of specimens (syntypic) from Koshun (= Hengchun), southern Taiwan. Both Andy Hamilton and Masami Hayashi (pers comm.) examined the type series in Entomological Institute, Hokkaido University, Sapporo, Japan (9 ♂♂ and 4 ♀♀) and the latter’s photographs of a syntype are reproduced here (Fig. 5C, D). One of the male syntypes had been dissected and bears a determination label by Andy Hamilton indicating the species belongs to *Amritodus*, which needs to be confirmed, together with an unpublished lectotype label. The label information on the specimens is as follows (hw = handwritten, pr = printed), double quotation marks show each label, a single oblique line (except between day and month) is used for changing to new line, and the description on underside is indicated after double oblique lines:

- 1 male [dissected], “Formosa / Matsumura [printed] // Koshun / 7/7-02 [hw]”, “ *I.formosanus* n.sp. [hw] / det. Matsumura [pr]”, “Type Matsumura” (red printed label), “Lectotype [red printed label] / *Idiocerusformosanus* Mm. [hw by K.G.A. Hamilton, not published]”, “ *Amritodusformosanus* (Matsum.) / Det. KGAH ’76” [in double red frame, hw]. 2 male, same data as previous, “PARATYPES [yellow printed label] / *Idiocerusformosanus* Mm. [hw]”
- 4 male, 3 female, [upper right male dissected], “Formosa / Matsumura [pr] // 7/VII 1906 | Koshun [hw]”, “Paratypes (yellow printed label) / *Idiocerusformosanus* Mm.” [hw];
- 1 female, “Formosa / Matsumura [pr] // 29/VI 1906 / Koshun (hw)”
- 1 male, “Formosa / Matsumura [pr] // 7/VII 1906 / Koshun [hw]
- 1 male, “Formosa / Matsumura [pr] // 10/VIII 1906 / Koshun [hw]”

## Supplementary Material

XML Treatment for Idioscopus

XML Treatment for Idioscopus bihamulus

XML Treatment for Idioscopus ventrispinus

XML Treatment for Idioscopus clypealis

XML Treatment for Idiocerus apicalis

XML Treatment for Idiocerus formosanus

## References

[B1] Baker CF (1915) Studies in Philippine Jassoidea. IV. The Idiocerini of the Philippines. Philippine Journal of Crop Science 10: 317–343.

[B2] Dietrich CH (2005) Keys to the families of Cicadomorpha and subfamilies and tribes of Cicadellidae (Hemiptera: Auchenorrhyncha). The Florida Entomologist 88(4): 502–517. 10.1653/0015-4040(2005)88[502:KTTFOC]2.0.CO;2

[B3] Huang KW Maldonado-Capriles J (1992) Idiocerinae of Taiwan (Homoptera: Cicadellidae). Journal of Taiwan Museum 45(1): 1–14.

[B4] Khatri I Webb MD (2014) Review of the idiocerine leafhoppers of Pakistan (Hemiptera, Cicadellidae) with a description of a new species. Zootaxa 3860(3): e280. 10.11646/zootaxa.3860.3.6 25283206

[B5] Kuoh ZL Fang QQ (1985) Two new species of *Idioscopus* from China (Hemiptera: Cicadellidae: Idiocerinae). Dong Wu Fen Lei Xue Bao 10(2): 189–192.

[B6] Lethierry LF (1889) Definitions of three new Homoptera. Journal of the Asiatic Society of Bengal 58: 252–253.

[B7] Maldonado-Capriles J (1964) Studies on Idiocerinae leafhoppers: II. The Indian and Philippine species of *Idiocerus* and the genus *Idioscopus* (Homoptera: Cicadellidae). Proceedings of the Entomological Society of Washington 66: 89–100.

[B8] Maldonado-Capriles J (1974) Studies on Idiocerinae leafhoppers XII. *Idioscopusclavosignatus* spec. nov. (Homoptera, Cicadellidae). Zoölogische Mededeelingen 48(15): 163–167.

[B9] Oman PW (1949) The Nearctic leafhoppers (Homoptera: Cicadellidae) a generic classification and check list. Memoirs of the Entomological Society of Washington 3: 1–253.

[B10] Pruthi HS (1936) Studies on Indian Jassidae (Homoptera). Part III. Description of some new genera and species, with first records of some known species from India. Memoirs of the Indian Museum 11: 101–131.

[B11] Viraktamath CA (1976) Four new species of Idiocerine leafhoppers from India with a note on male *Balochaastute* (Melichar) (Homoptera: Cicadellidae: Idiocerniae). Mysore Journal of Agricultural Sciences 10: 234–244. 10.1080/00305316.1976.10432323

[B12] Viraktamath CA (1979a) *Jogocerus* gen. nov. and new species of idiocerine leafhoppers from southern India (Homoptera: Cicadellidae). Entomon 4(1): 17–26.

[B13] Viraktamath CA (1979b) Four new species of *Idioscopus* (Homoptera: Cicadellidae) from Southern India. Entomon 4(2): 173–181.

[B14] Viraktamath CA (1980) Notes on *Idioscopus* species (Homoptera: Cicadellidae) described by Dr. H.S. Pruthi, with description of a new species from Meghalaya, India. Entomon 5(3): 227–231.

[B15] Viraktamath CA (1989) Auchenorrhyncha (Homoptera) associated with mango, *Mangiferaindica* L. Tropical Pest Management 35(4): 431–434. 10.1080/09670878909371423

[B16] Walker F (1869) Catalogue of the Homopterous insects collected in the Indian Archipelago by Mr. A.R. Wallace, with descriptions of new species. Zoological Journal of the Linnean Society 10(45): 276–330. 10.1111/j.1096-3642.1869.tb00663.x

[B17] Wang XY Dai RH (2018) A new species of *Idioscopus* harming *Myricarubra* (Hemiptera: Cicadellidae: Idiocerinae). Journal of Mountain Agriculture and Biology 37(6): 10–13. [in Chinese]

[B18] Wang XY Wang JJ Zhou XL Dai RH (2021) Description of three new species of the leafhopper genus *Idioscopus* Baker, (Hemiptera: Cicadellidae: Eurymelinae) from Yunnan, China. Zootaxa 4995(2): 375–381. 10.11646/zootaxa.4995.2.10 34810564

[B19] Xue QQ Viraktamath CA Zhang YL (2017) Checklist to Chinese Idiocerine leafhoppers, key to genera and description of a new species of *Anidiocerus* (Hemiptera: Auchenorrhyncha: Cicadellidae). Entomologica Americana 122(3): 405–417. 10.1664/1947-5144-122.3.405

[B20] Zhang B Li Z (2012) Idiocerinae. In: Dai L Li Z Jin D (Eds) Insects from Kuankuoshui Landscape.Guizhou Science and Technology Press, Guiyang, 202–209.

